# Regional Pulmonary Ventilation Assessment Method and System Based on Impedance Sensing Information from the Pentapulmonary Lobes

**DOI:** 10.3390/s24103202

**Published:** 2024-05-17

**Authors:** Yapeng Zhang, Chengxin Song, Wei He, Qian Zhang, Pengcheng Zhao, Jingang Wang

**Affiliations:** State Key Laboratory of Power Transmission Equipment Technology, School of Electrical Engineering, Chongqing University, Chongqing 400044, China; 202111021157t@stu.cqu.edu.cn (Y.Z.); 20173598@cqu.edu.cn (C.S.); hewei@cqu.edu.cn (W.H.); zhangqian@cqu.edu.cn (Q.Z.); zhaopengcheng@cqu.edu.cn (P.Z.)

**Keywords:** regional ventilation information, biosensor, pulmonary function parameters, ventilation disorders

## Abstract

Regional lung ventilation assessment is a critical tool for the early detection of lung diseases and postoperative evaluation. Biosensor-based impedance measurements, known for their non-invasive nature, among other benefits, have garnered significant attention compared to traditional detection methods that utilize pressure sensors. However, solely utilizing overall thoracic impedance fails to accurately capture changes in regional lung air volume. This study introduces an assessment method for lung ventilation that utilizes impedance data from the five lobes, develops a nonlinear model correlating regional impedance with lung air volume, and formulates an approach to identify regional ventilation obstructions based on impedance variations in affected areas. The electrode configuration for the five lung lobes was established through numerical simulations, revealing a power–function nonlinear relationship between regional impedance and air volume changes. An analysis of 389 pulmonary function tests refined the equations for calculating pulmonary function parameters, taking into account individual differences. Validation tests on 30 cases indicated maximum relative errors of 0.82% for FVC and 0.98% for FEV1, all within the 95% confidence intervals. The index for assessing regional ventilation impairment was corroborated by CT scans in 50 critical care cases, with 10 validation trials showing agreement with CT lesion localization results.

## 1. Introduction

In the post-epidemic era, regional lung diseases such as pneumonia have seriously jeopardized people’s lives and health and are widely seen in populations of critically ill patients [1]. The accurate assessment and management of the local ventilation status of the lungs is critical for the postoperative monitoring of critically ill patients and early identification screening of normal individuals [2]. Imaging is used as a primary means of regional lung ventilation assessment, utilizing sensors such as X-ray detectors and radiofrequency receivers to obtain lung image information [3]. Its application is limited by a specific detection environment and cannot provide continuous lung ventilation information in real time. Pulmonary ventilation parameter support is widely used in clinical practice because of its portability and rapidity. FVC and FEV1 are used to assess the level of pulmonary ventilation as the main evaluation parameters of lung health status [4,5]. Conventional pulmonary function testing uses flow rate sensors and pressure sensors to measure gas flow at the respiratory passages, which, in turn, indirectly assesses changes in lung air volume [6]. Pulmonary ventilation parameters are widely used in clinical practice. It is not suitable for critically ill patients because of the large expiratory resistance in its measurement process [7], and it cannot provide regional ventilation parameters. Bioimpedance technology is based on biosensors for physiological measurements and enables non-invasive ventilation assessment [8,9,10,11]. However, current bioimpedance-based lung ventilation assessment methods rely on overall thoracic impedance to infer lung air volume changes, resulting in low accuracy of ventilation parameter calculations and failing to consider the relationship between regional impedance and local lung ventilation.

Biosensing technology employs biomaterials and sensors to capture biological data from tissues and organs, transforming these data into clinically relevant information that reflects the physiological status of the human body [12]. The effectiveness of using biosensors for measuring body impedance to identify internal structural abnormalities in the human body has been well documented [13,14,15,16]. Given the significant changes in medium and tissue structure within the lungs during respiration, bioelectrical impedance techniques have increasingly been applied to evaluate lung ventilation [17,18,19]. A study referenced in [20] investigated lung impedance under continuous ventilation using an impedance tracing method, establishing a link between lung impedance and ventilation function. Further, research in [21] identified a nonlinear mathematical relationship between lung impedance and ventilation via experimental studies. To develop a model for calculating lung function parameters, Yang et al. created a four-electrode thoracic impedance measurement device and proposed a method based on controlled experimental data [22]. Zhao Pengcheng et al. extended this work by calculating lung function parameters through thoracic impedance measurements in two orthogonal directions [23]. However, due to the thoracic contour’s undulation and the non-uniform flow of fluids within the thoracic cavity during respiration, total thoracic cavity impedance changes encompass both lung air volume changes and impedance shifts in other thoracic media. Consequently, models based on overall impedance inaccurately estimate lung air volume changes, failing to capture regional variations.

The research documented in reference [24] indicates significant variability in bioimpedance measurements across different electrode configurations. To derive bioimpedance coefficients that more accurately reflect local lung lesion characteristics and to develop a more precise calculation model, reference [25] first analyzed the sensitivity of impedance measurements using a four-electrode setup, establishing a benchmark for targeted impedance analysis. Subsequently, reference [26] demonstrated the targeted measurement of pulmonary edema through a specific electrode configuration, further validating the efficacy of focused impedance measurement. Reference [27] integrated focused impedance techniques with traditional spirometry to facilitate the straightforward evaluation of restrictive lung ventilation disorders. Additionally, reference [28] explored the depth sensitivity of impedance measurements in lung tissues using a six-electrode model, suggesting that focused impedance measurements in specific lung regions can be achieved by designing an appropriate driven measurement model.

In order to improve the accuracy of the calculation of pulmonary function parameters and to realize the assessment of regional lung ventilation status based on impedance sensing information using biosensors, the five lobes of the lungs were used as the ROI, and the impedance focus evaluation method of the region of interest was studied, and a method of calculating pulmonary function parameters based on impedance information of the five lobes and a method of assessing the regional lung ventilation status were proposed. The advantage of this method is that it makes full use of the different electrode configurations for different lung regions of interest by designing the five electrode patterns with the highest degree of interest for the five lung lobes, enabling the focused measurement of impedance changes in each lung region. In addition, the coefficient matrix of the pulmonary function parameter calculation model is calibrated according to the test data of the pulmonary function department, and the coefficient matrix is optimized based on the individual difference parameters, which makes the calculation model universal and effectively improves the calculation accuracy. Based on the clinical data of critically ill patients, the evaluation index of regional ventilation obstruction in five lung lobes was determined to provide clinical support for regional ventilation information.

Therefore, the work in this paper focuses on the following points:

- The design of an impedance-focusing evaluation method for thoracic ROI, deriving and validating the model using different electrode configurations with an interest in evaluating the impedance change in five lung lobes, and proposing an interest-focusing measurement method for five lung lobes accordingly.
- The construction of a 2-D longitudinal simulation model of the thoracic cavity, qualitatively analyzing the most interesting electrode configuration combinations for the five lung lobes. The 3-D simulation model of the lung was reconstructed to quantitatively analyze and verify the feasibility of the quadrant measurement mode, and the mathematical calculation model between air volume and zonal impedance was initially constructed based on the numerical results of the simulation.
- Based on the ADS1292, a biosensor analog front-end developed by Texas Instruments, a multi-channel bio-impedance acquisition system was designed, 389 subjects were selected to carry out lung function parameter testing, and the calculation method was established based on the optimized calculation model of individual influence coefficients. Then, 30 subjects were selected to carry out experimental validation, and the results showed that the calculated values of this method were in good agreement with the standard values, with small errors, and had better accuracy than other impedance measurement methods.
- The regional lung ventilation obstruction evaluation test was conducted by selecting 50 critically ill patients with localized lung ventilation obstruction, and the ventilation obstruction evaluation index was obtained. Ten more critically ill patients were selected to carry out the test validation, and the results showed that the evaluation method was consistent with the evaluation results of CT images and could provide auxiliary guidance for clinical diagnosis.

## 2. Methods for Evaluating the Region of Interest of the Pentapulmonary Lobes

**Figure 1:**
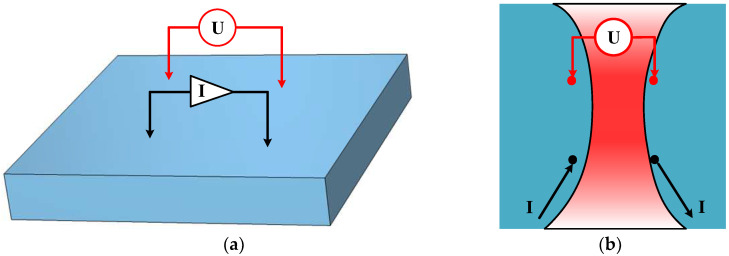
Four-electrode square configuration and visualization of ROI. (**a**) Four-electrode square configuration; (**b**) isopotential distribution in the ROI.

Human body impedance measurements use the body as a current conductor by injecting current diffused in all directions into the body from body surface excitation electrodes. The current in the body converges to the high conductivity region and flows through to the measuring electrodes to form the specific potential, which is used to characterize changes in the internal physiology of the body by calculating impedance changes. Due to the non-homogeneous nature of tissues within the human body, changing the excitation measurement pattern restricts the flow of excitation current through a specific region and focuses on measuring impedance changes in specific regions of the body to assess region-specific pathological changes. Currently, the most common electrode configuration for human thoracic impedance measurement is the four-electrode square structure shown in Figure 1a, in which the electrodes are placed on the thoracic body surface in a square, symmetrical pattern to realize impedance-focusing measurements in the square area. The red region between the two equipotential lines crossing the electrode in Figure 1b represents the region of interest (ROI) for this configuration. This means that the impedance changes measured by this electrode configuration are most sensitive to conductivity changes in the ROI and that impedance-focused measurements in the ROI can be realized by designing specific electrode configurations.

The structure of the human thoracic cavity is highly inhomogeneous and anisotropic. The complex internal organization poses great difficulties for the convergence of numerical analysis in 3-D simulations. Because the body surface excitation current forms spindle-like current fields inside the thoracic cavity, the effect of the normal direction can be neglected for deeper ranges and regions with small differences in cross-sectional conductivity distributions. Therefore, the 3-D field calculation problem can be simplified to a 2-D field analysis to assess the ROI with the ability to measure impedance focusing in 2-D longitudinal sections of the thoracic cavity.

**Figure 2:**
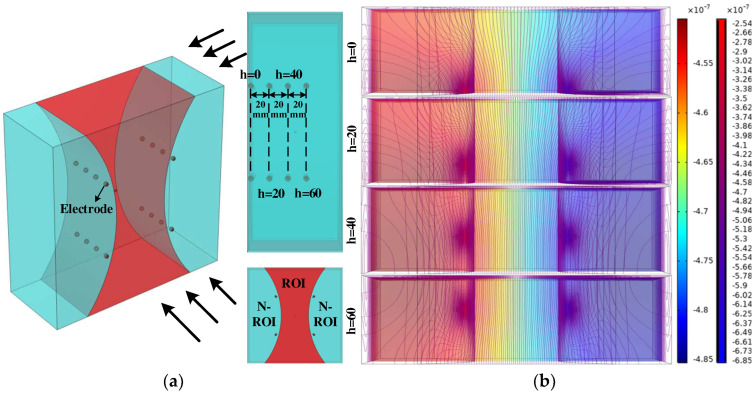
Three-dimensional longitudinal−like thoracic cavity modeling and simulation. (**a**) Three-dimensional longitudinal−like thoracic cavity modeling; (**b**) potential and equipotential line distribution.

In order to further validate the above conclusions, the 3-D longitudinal thorax-like model, as shown in Figure 2a, is established in this article, and the field is partitioned into ROI and non-interested regions (N-ROIs) according to the focusing characteristics of the four-electrode square configuration, and the impedance changes in the specific regions are simulated and analyzed (see Section 3 for the simulation parameters).

The potential and isopotential line distributions derived from the simulation in Figure 2b show that for the same electrode arrangement pattern placed within the longitudinal section of the thoracic cavity at different depths h, the potential distribution of the red ROI varies very little in the longitudinal section normal to the square and the isopotential lines in this region are distributed perpendicular to the longitudinal section. Thus, the tangent direction in the longitudinal section is the main potential change direction. In addition, the simulation results of the model at different depths h lead to the same conclusion. Then, the potential changes between the measurement electrodes with the same electrode arrangement on the body surface at the corresponding position of the chest cavity in the 3-D model are capable of being characterized by analyzing the 2-D longitudinal section model of the chest cavity.

**Figure 3:**
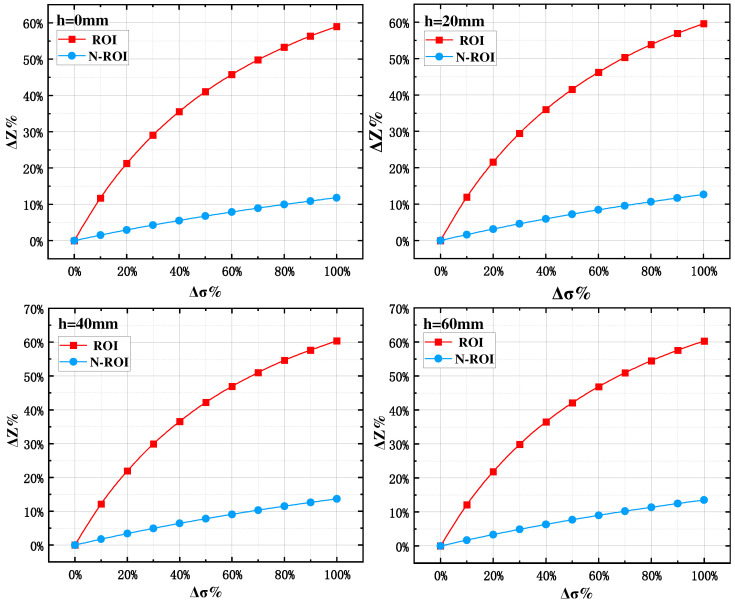
Impedance versus conductivity rate of change curves at different depths h.

In order to comparatively analyze the focusing effect of impedance measurements on the ROI with the same electrode arrangement at different longitudinal section depths, the rate of change of conductivity in each region was synchronously recorded with the rate of change of the potential difference of the measuring electrodes, as shown in Figure 3. When the ROI and N-ROI generate the same rate of change in conductivity, the ROI has the largest rate of change in boundary potential. Therefore, the red region is the ROI in this electrode mode. In addition, the boundary potential rate of change curves of ROI and N-ROI are approximately the same when the electrodes are placed in the longitudinal section at four different depths h. The boundary potential change rate curves of ROI and N-ROI are approximately the same. This means that there is no significant difference in the focusing measurement pattern of the ROI obtained by any 2-D thoracic longitudinal section simulation, and it is possible to provide guidance for body surface electrode deployment by 2-D longitudinal section simulation.

When the traditional four-electrode method is used to measure bioelectrical impedance to characterize ventilation parameters, the boundaries of the region of interest range are unclear, and the computational model of ventilation parameters is constructed by the equivalent impedance in only one direction, which is associated with large nonlinearity. Based on the traditional four-electrode method, a lung-specific ROI measurement and evaluation method is proposed to provide ventilation parameters containing impedance information of the penta-lobe region by designing the driven measurement mode of the electrodes.

The nature of the ROI for the particular electrode mode is defined as the change in potential of the measuring electrode being most sensitive to the change in conductivity in the ROI. The longitudinal section of the lung is uniformly partitioned into *x* discrete regional units, and for *y* electrode patterns, the conductivity *σ* and the measured voltage *v* of all discrete units can form the mathematical relationship shown in (1):

(1) $$\left\{ \begin{array}{l} v_{1}=w_{1}(\sigma_{1},\sigma_{2},\sigma_{3},\cdots \sigma_{x})\\ v_{2}=w_{2}(\sigma_{1},\sigma_{2},\sigma_{3},\cdots \sigma_{x})\\ \;\;\;\;\;\;\;\;\;\;\;\;\;\;\;\;\cdots \\ v_{y}=w_{y}(\sigma_{1},\sigma_{2},\sigma_{3},\cdots \sigma_{x}) \end{array}\right.$$

Defining the above mapping relationship between the x-dimensional conductivity vector and the y-dimensional measured voltage vector as *W*, the nonlinear relationship between the potential values and the conductivity distribution of the thoracic body surface measurement electrodes can be described as follows:

(2) V=W(σ)

where *V* in (2) characterizes the boundary voltage measurement at the measurement electrode, and σ is the conductivity distribution inside the chest cavity. Then, the Taylor expansion of (2) at *σ* = *σ*_0_ is

(3) $$V=V_{0}+{\frac{dW(\sigma )}{d\sigma }|}_{\sigma =\sigma_{0}}(\sigma -\sigma_{0})+O[{\left(\sigma -\sigma_{0}\right)}^{2}]$$

When a slight perturbation of the internal conductivity of the chest cavity occurs, the quadratic term in (3) can be approximately neglected, and then the expression for the change in voltage measured at the boundary at this point can be simplified as follows:

(4) $$\Delta V=V-V_{0}\approx {\frac{dW(\sigma )}{d\sigma }|}_{\sigma =\sigma_{0}}\Delta \sigma$$

Discretizing (4) to obtain the relationship between small changes in conductivity Δ*σ* and measured voltage in the discrete region:

(5) $$S=\frac{\partial V}{\partial \sigma }$$

where *S* is the *x* × *y* evaluation matrix of interest:

(6) $$S=\left[\begin{array}{ccc} \frac{\partial V_{1}}{\partial \sigma_{1}} & \cdots & \frac{\partial V_{1}}{\partial \sigma_{x}}\\ \vdots & \ddots & \vdots \\ \frac{\partial V_{y}}{\partial \sigma_{1}} & \cdots & \frac{\partial V_{y}}{\partial \sigma_{x}} \end{array}\right]$$

The magnitude of the value of the *S*_ij_ element in the *i*-th row and *j*-th column of the evaluation matrix of interest *S* indicates the gradient of the potential change of the measurement electrode for the *i*-th electrode configuration when the conductivity of the area cell labeled *j* is changed slightly. If the amplitude of an element of the evaluation matrix of interest is large, it means that that particular discrete region space is the ROI for the current measurement pattern.

There are similarities in the derivation of the evaluation matrix of interest *S* proposed in this paper and the sensitivity matrix utilized in Electrical Impedance Tomography (EIT), both focusing on the relationship between the change in conductivity and boundary potential in the field. However, there is a fundamental difference between the methodology of this paper and EIT in the use of this matrix. The research in this paper focuses on exploring the relationship between discrete changes in conductivity and corresponding changes in boundary potentials within a specific region, with the aim of developing a focused measurement method suitable for lung partitioning rather than lung image reconstruction, as investigated using EIT. While the underlying calculations may appear similar to those used in EIT, the application of our derived coefficients specifically addresses discrete regional analysis rather than full-field image reconstruction.

**Figure 4:**
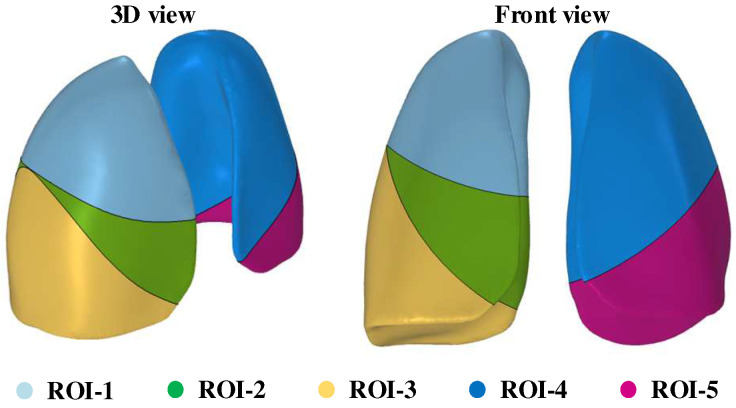
Three-dimensional modeling of pentapulmonary lobes of interest.

Figure 4 shows the 3-D modeling of pentapulmonary lobes of interest designed in this article. Different color regions characterize the set of regional units of a specific lung lobe. The five lung lobes are treated as five regions of interest, respectively, and in order to evaluate the optimal measurement method for each *ROI*, the coefficient of interest *P* is proposed, and the coefficient of interest *P_ROI_* for a specific *ROI* can be expressed as follows:

(7) $$P_{ROI}=\sum_{j\;\in \;\Omega_{ROI}}\left|S_{ij}\right|$$

where Ω*_ROI_* is the set of all discrete regional units within the *ROI*. A larger value of the coefficient of interest *P_ROI_* means that the ith measurement mode is more sensitive to perceiving the ventilation changes in the *ROI*. In contrast to the traditional EIT approach to sensitivity matrices by regularization, this paper evaluates impedance-focused measurements of any combination of discrete regions of the lung by (7). The fundamental reason for adopting this evaluation method the focus of this article is to assess the sensitivity of region-specific conductivity changes in a defined electrode stimulation pattern rather than to perform extensive image reconstruction of a region. The method greatly simplifies the computational model and reduces the effect of noise in less relevant regions.

Based on the results of the simulated numerical studies in Section 3, the electrode mode of most interest for each of the five lung lobes was determined, and the impedance change and airflow change for each lung lobe satisfy a specific nonlinear relationship. In this article, this nonlinear relationship is defined as *O*. Then, the change in lung ventilation Δ*L* for each *ROI* satisfies the following multivariate nonlinear relationship:

(8) $$\Delta L_{ROI-i}\;\;=O(\Delta Z_{ROI-i}),i\in \{1,2,3,4,5\}$$

**Figure 5:**
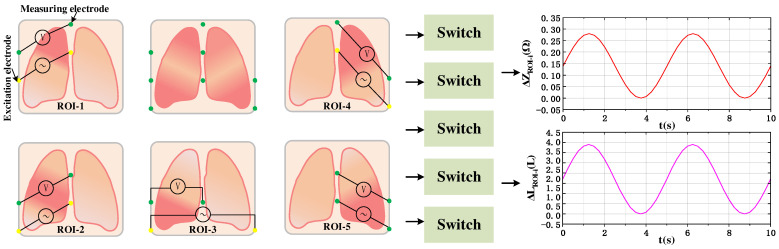
Procedure for calculating regional lung ventilation in five lobes.

Figure 5 shows the process of regional impedance measurement and air volume change calculation in five lung lobes. By switching to the specific lung region to focus the measurement mode through the analog switch to realize the simultaneous measurement of the impedance change volume of the specific lung lobe, and then calculate the lung function parameters according to the regional ventilation change volume calculated using Equation (8).

## 3. Simulation Studies and Regional Lung Ventilation Assessment Method

### 3.1. Simulation Theory and Modeling

The real-time measurement of thoracic impedance is mainly performed by injecting a low-frequency AC current of 64 kHz into the body by means of the excitation electrodes on the body surface. Based on the scattering frequency theory of bioelectrical impedance, each biological tissue is capable of neglecting the effect of displacement current under low-frequency excitation, and this sinusoidal time-varying electromagnetic field is approximated as a magnetic quasi-static field. Then, the Poisson equation for the forward solution of the simulation study is as follows:

(9) $$\nabla^{2}\varphi =-\frac{\nabla \sigma \cdot \nabla \varphi }{\sigma }$$

From (9), it is shown that changes in tissue conductivity and conductivity distribution within the thoracic cavity are capable of inducing changes in the boundary measurement voltage *φ*.

The excitation current is set to 1 mA during the simulation, the current injection place is assumed to be uniformly distributed, and the boundary potential at the non-current injection place is consistent with the distribution within the thoracic field, then the normal current density *Jn* at the boundary target is satisfied:

(10) $$J_{n}=\left\{ \begin{array}{l} 1\;\;\mathrm{mA}/S,\mathrm{Current}\;\mathrm{injection}\;\mathrm{place}\\ 0,\;\;\;\;\;\;\;\;\;\mathrm{Non}-\mathrm{current}\;\mathrm{injection}\;\mathrm{place} \end{array}\right.$$

**Figure 6:**
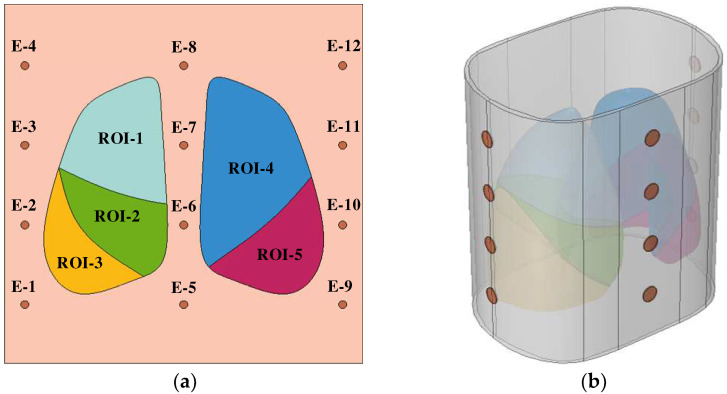
Simulation model. (**a**) Two-dimensional longitudinal modeling of the thoracic cavity; (**b**) Three-dimensional thoracic model.

To further investigate the optimal electrode pattern using pentapulmonary lobes as the ROI, a 2-D thoracic longitudinal model, as shown in Figure 6a, was constructed for this qualitative simulation study. Based on the electrode mode determined by the 2-D simulation, the 3-D thoracic cavity model shown in Figure 6b was constructed and the numerical study was carried out by using finite element simulation software.

A previous study [29] showed a negative correlation between lung tissue conductivity and lung gas filling coefficient *n*, which is the ratio of lung gas volume to total lung volume, as shown in (11). Considering the uncertainty of the lung contour change during human breathing, the human thoracic impedance change under the dynamic breathing change is mainly studied equivalently by the conductivity parameter change during breathing in the simulation process.

(11) $$\sigma_{Lung}\propto \frac{k}{n^{0.1942}}$$

where *σ_Lung_* represents the conductivity of the lungs, and *k* is the conductivity scaling factor.

To further determine the conductivity scaling factor k, the conductivity of the lung tissue samples was measured based on a bioimpedance analyzer at an excitation frequency of 64 kHz to obtain a lung conductivity function based on the experimental data:

(12) $$\sigma_{Lung}=\frac{0.1802}{n^{0.1942}}$$

In order to make the simulation results more accurate and reliable, the corresponding conductivity is set for each tissue of the refined model during the simulation process, and the specific design of human tissue conductivity parameters is shown in the following Table 1:

### 3.2. Qualitative Study Based on 2-D Longitudinal Thoracic Section Modeling

**Figure 7:**
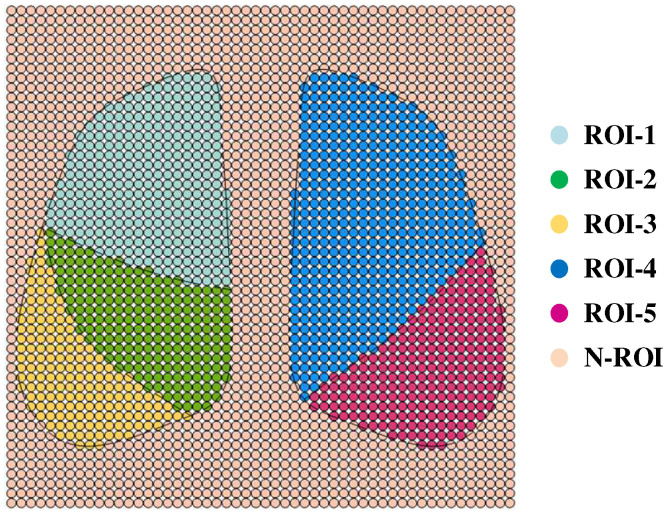
A discrete network of regions in a two-dimensional thoracic model.

To evaluate the regional impedance focusing level, the 2-D thoracic longitudinal section model was partitioned into a grid of discrete cells, as shown in Figure 7, and the whole lung region was discretized into a total of 2704 regions. In order to fully investigate the optimal electrode configuration using the pentapulmonary lobe as the ROI, all electrode combinations consisting of 12 electrodes were numerically analyzed in this simulation. To reduce the influence of human contact impedance, the excitation electrode and the measurement electrode need to be separated, leading to a total of C^2^_12_ × C^2^_10_ = 2970 electrode modes. The degree of interest of each electrode mode on different lung lobes was solved via simulation to provide the basis for the subsequent 3-D numerical study.

Based on the conductivity of each region set in Table 1, the evaluation matrix of interest S is calculated by (6). The resulting S-matrix is a matrix of 2970 rows and 2704 columns, and each row element in the matrix characterizes the level of interest of the electrode pattern corresponding to all discrete regions of the lung.

**Figure 8:**
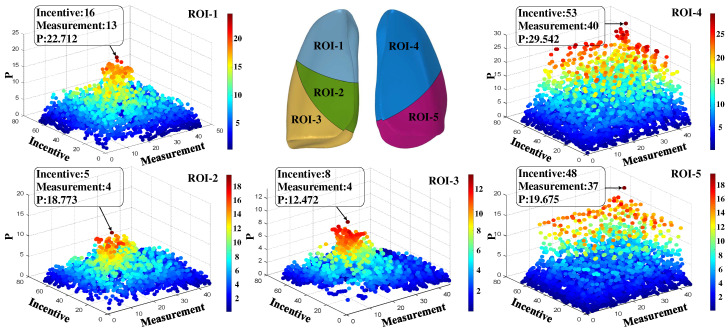
Distribution of coefficients of interest for pentapulmonary lobes.

To facilitate the analysis of ROI for specific electrode modes, all electrode modes were processed with matrix labeling. The 2970 electrode modes can be divided into C^2^_12_ = 66 excitation electrode configurations, and each electrode configuration contains C^2^_10_ = 45 measurement electrode configurations under each electrode configuration, then the 2970-dimensional evaluation coefficient column vectors of interest computed for a specific lung region can be transformed into a 66 × 45 evaluation matrix. Where 66 rows of the evaluation matrix characterize the number of all combinations formed by selecting 2 non-identical electrodes for the 12 electrodes, and 45 columns represent the number of all combinations formed by selecting two electrodes out of the ten electrodes, excluding the two excitation electrodes. Both permutation traversal modes use pairing of the least-labeled element with each subsequent element one at a time in ascending order. The matrix of evaluation coefficients of interest for each mode for the 2970 × 5 five lung lobes was calculated according to (7), and each column was processed as described above to obtain five 66 × 45 evaluation matrices, the distribution of which is shown in Figure 8.

From the definition of (7), when the coefficient of interest is maximized, then the corresponding lung region is the ROI for that electrode mode. The three electrodes in equidistant rows from the right thoracic cavity to the left side of the lowermost layer were labeled sequentially as electrode No. 1, electrode No. 5, and electrode No. 9, with each column of electrodes labeled from the bottom upwards in ascending order. The optimal electrode configurations for the five lung lobes as ROI were determined based on the location of the largest element of the five lung lobes of interest evaluation matrix shown in Figure 8, as shown in Table 2. Then, it was determined that electrodes 2 and 7 were for excitation, electrodes 3 and 8 were for measurement, and the upper lobe of the right lung was the ROI. Electrodes 1 and 6 were excited, electrodes 2 and 7 measured, and the middle lobe of the right lung was the ROI. Electrodes 1 and 9 were for excitation, electrodes 2 and 6 were for measurements, and the lower lobe of the right lung is the ROI. Electrodes 7 and 9 were for excitation, electrodes 8 and 10 were for measurements, with the upper lobe of the left lung as the ROI. Electrodes 2 and 6 were for measurements, with the lower lobe of the right lung as the ROI. Electrodes 6 and 9 were excited and electrodes 7 and 10 measured with the upper lobe of the left lung as the ROI. Subsequent quantitative studies of 3-D modeling followed these measurement modes of research.

In addition, based on the distribution of the coefficients of interest in each electrode mode shown in Figure 8, it can be seen that for the five specific lung zones, in addition to the optimal electrode modes, other electrode modes with higher sensitivity coefficients also exist. Since the goal of the study in this article is to achieve an optimal balance between measurement accuracy and clinical utility, the eight electrodes using the setup shown in Table 2 were able to provide sufficient resolution for focused measurements in the five specified lung zones. Adding other measurement modes would surely increase the number of electrodes, which introduces greater complexity, increases setup time, and may cause patient discomfort. Therefore, this paper discusses and investigates the 8-electrode configuration described above.

**Figure 9:**
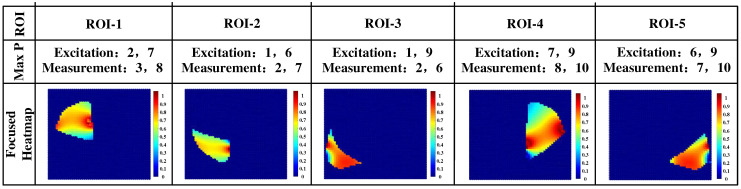
Thermogram of interest of the five lung lobes in the optimal electrode configuration.

To further demonstrate the feasibility of using the coefficient of interest as an evaluation index, the corresponding heat map of the region of interest under each configuration was plotted according to the obtained optimal electrode configurations of the pentapulmonary lobes, as shown in Figure 9.

### 3.3. Quantitative Study Based on 3-D Thoracic Modeling

Considering that the respiratory process of the subject was deep breathing, the range of variation in the gas filling coefficient of the lungs was selected as *n* ϵ (0.2~0.4). To obtain the mathematical relationship between the change in gas volume and the amount of impedance change in the five lobes of the lungs during the respiratory process, a parameterized scanning simulation was performed on the gas filling coefficient using the 3-D five-lobe model, and the impedance measurements were recorded for the five electrodes modes when the gas volume changed individually within the five lobes, as shown in Table 3. Z_i_ denotes the impedance value of the ith ROI region at different respiratory states, respectively. ΔZ_i_% denotes the rate of change of impedance at the end of inspiration with the beginning of inspiration for the ith ROI region, respectively.

**Figure 10:**
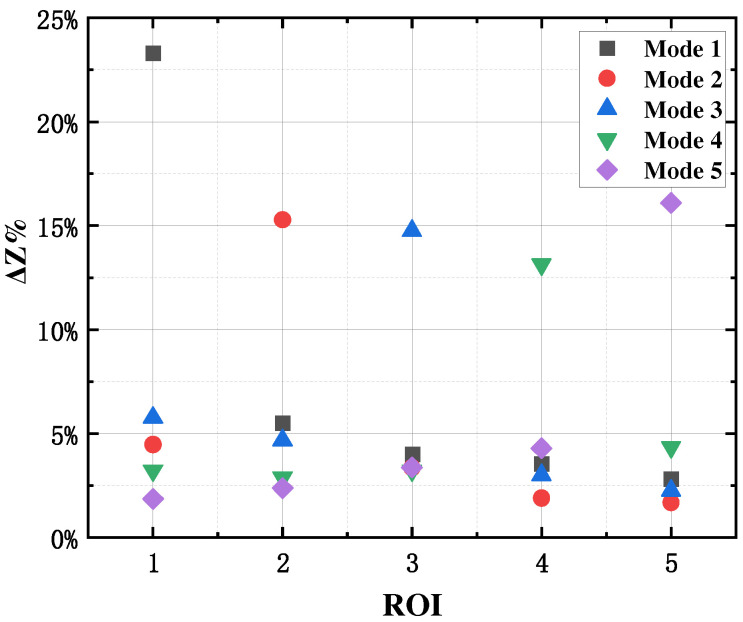
Rate of change in impedance in pentapulmonary lobes with optimal electrode configuration.

The maximum rate of change in measured impedance in the ROI indicates the degree of impedance change during inspiration. It is capable of indirectly characterizing the perceived sensitivity of this measurement mode to gas changes in a specific lung region. Figure 10 shows the maximum rate of change of impedance for the five optimal electrode configurations when the same air volume change occurs in the five lung lobes. It can be seen that each electrode configuration has the largest value of the maximum rate of change of impedance for its ROI, further demonstrating the feasibility of the optimal electrode design for the five lung lobes.

In addition, Figure 10 also indicates that the optimal electrode configuration for a specific lung lobe also has a sensitivity of about 5% to impedance changes caused by changes in air volume in other lung lobes. This means that the optimal electrode configuration for a specific lung region may be affected by changes in air volume in other lung regions. Since the fundamental purpose of designing a specific biofocused measurement method is to seek the relatively most sensitive electrode pattern, it is unrealistic to achieve a fully focused measurement. Moreover, for a given lung region, the impedance information measured by other electrode modes contains much less information about air volume changes in that lung region than in other lung regions. Therefore, in the subsequent study, the impedance measurement data of one optimal electrode mode were used to construct a mathematical model for air volume calculation in a specific lung region.

**Figure 11:**
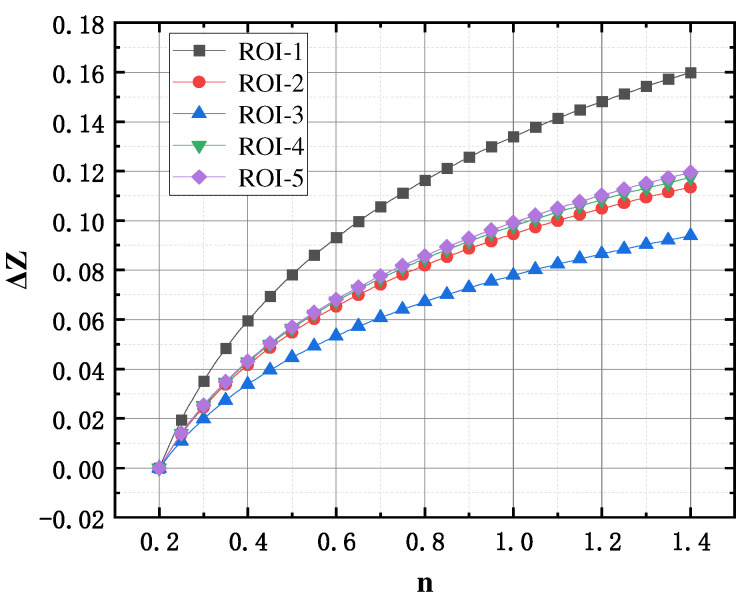
Curves of impedance changes in each lung lobe versus air volume in the lung area.

When the human body completes a respiratory cycle, the main medium of change within the thoracic cavity is air. The impedance change amount Δ*Z_i_* of each lung lobe can characterize the amount of regional gas change to a certain extent. According to Table 3, the impedance change volume versus air volume curve shown in Figure 11 was obtained, which shows that the larger the gas volume change in the lungs of the subject, the larger the bioelectrical impedance change in the lungs. In addition, there is a strong positive correlation between the amount of regional lung impedance change caused by breathing and the amount of gas volume change. This is because the main medium of change in the body during respiration is the change of air in the lungs, and it can be approximated that there is a definite mathematical relationship between the change in pulmonary impedance and the change in lung gas volume.

Loss of gas exchange function in localized regions of the lungs is the most distinctive feature of localized ventilation dysfunction diseases, such as localized pulmonary fibrosis, pulmonary embolism, and emphysema. To further obtain information on regional lung lesions in five lobes, localized lesion studies were carried out by calibrating the amount of impedance change in specific lobes of the 3-D lung model to zero.

**Figure 12:**
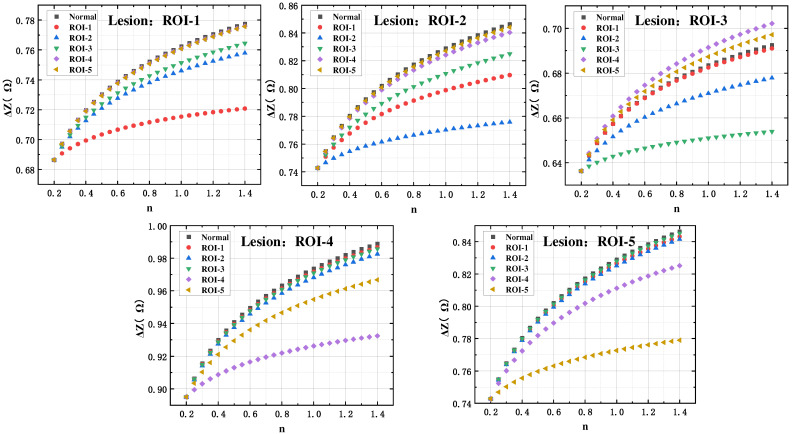
Curve of the amount of impedance change in the five lung lobes under regional lung lesions.

The impedance change volume curves measured using the five electrode configurations when localized lesions occurred in the pentapulmonary lobes were obtained via 3-D lung model simulation, as shown in Figure 12. It can be seen that when a ventilation disorder occurs in a specific lung lobe region, the amount of impedance change measured by the electrode configurations using this region as the ROI is significantly lower compared with normal ventilation. This phenomenon is consistent with the air volume changes characteristic of localized regional lesions in the lungs. Therefore, regional lung ventilation dysfunction was subsequently assessed and simply calibrated by characterizing the amplitude change of the impedance change volume in each lung region.

### 3.4. Design of a Methodology for the Assessment of Ventilatory Function in Pentapulmonary Lobes

In order to further obtain the mathematical model of the impedance change volume of the five lung lobes and the gas change volume of each lung lobe, the curve data in Figure 11 were nonlinearly fitted, and it was found that the power function between the regional impedance change volume of the five lung lobes, Δ*Z_i_*, and the gas change volume of the various groups of lung areas, Δ*L_i_*, fitted best, all satisfying (13), and the R^2^ were 0.09956, 0.9927, 0.9965, 0.9982, 0.9974, and 0.9974, and R^2^ were all greater than 0.99, which were good fits.

(13) $$\Delta L_{i}=a_{i}\times {\left(\Delta Z_{i}\right)}^{b_{i}}+c_{i}$$

where *a_i_* denotes the multiplicative coefficient, *b_i_* denotes the power coefficient, and *c_i_* denotes the constant coefficient.

According to (13), five sets of [*a_i_ b_i_ c_i_*] coefficient vectors exist for the calculation of the amount of gas change in a specific lung region of the five lung lobes. Then, the coefficient matrix *A* is defined as follows:

(14) $$A=\left[\begin{array}{ccc} a_{1} & b_{1} & c_{1}\\ a_{2} & b_{2} & c_{2}\\ a_{3} & b_{3} & c_{3}\\ a_{4} & b_{4} & c_{4}\\ a_{5} & b_{5} & c_{5} \end{array}\right]$$

The simulated data derived from the simulation only reflect the generalized mathematical relationship between the amount of gas change in the lungs and the amount of regional impedance change, and the optimal characteristic coefficient matrix *A* was subsequently determined from the clinical trial data presented in Section 4.

**Figure 13:**
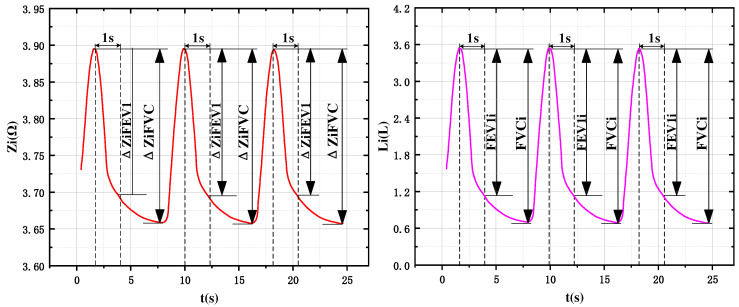
Waveform curves of regional impedance versus air volume over time (including interpretation of FVCi and FEV1i parameters).

Under the deep breathing state, the impedance information of five lung lobes was measured continuously, and the impedance waveform curves shown in Figure 13 were obtained for each lung region. Where Δ*Z_iFVC_* is the difference between the maximum impedance and minimum impedance in one respiratory cycle of the impedance curve of ROI-i, and Δ*Z_iFEV1_* is the value of impedance change at the end of inhalation and at 1 s of exhalation in one respiratory cycle of the impedance curve of ROI-i. Defining *FVCi* and *FEV*1i as the regional exertion lung volume and one-second exertion expiratory volume of ROI-i, substituting the above two parameters into (13), we were able to calculate *FVCi* and *FEV*1i used to characterize the ventilatory capacity of ROI-i.

(15) $$\left\{ \begin{array}{l} FVC_{i}\;\;\;\;=a_{i}\times {\left(\Delta Z_{iFVC}\;\;\right)}^{b_{i}}+c_{i}\\ FEV1_{i}=a_{i}\times {\left(\Delta Z_{iFEV1}\right)}^{b_{i}}+c_{i} \end{array}\right.$$

Conventional pulmonary function parameter testing instruments are not yet equipped to provide ventilation parameters for lung regions, and imaging devices only provide a qualitative assessment of ventilation impairment in lung regions. Therefore, the evaluation of the method in this paper adopts the medical lung function testing instrument as a benchmark instrument and compares the ventilation parameters of the whole lung region to verify the consistency. Then, the pulmonary function parameters of the five lung lobes are calculated using (15), and the ventilation parameters of the whole lung area can be obtained via (16).

(16) $$\left\{ \begin{array}{l} FVC\;\;\;\;=\sum_{i\in \{1,2,3,4,5\}}FVC_{i}\\ FEV1=\sum_{i\in \{1,2,3,4,5\}}FEV1_{i} \end{array}\right.$$

In order to further design an assessment method for establishing pentapulmonary lobes pathology, the normal ventilation of each lobe needs to be calibrated. The contribution of different lung lobes to lung ventilation is more consistent during the completion of respiration in normal lungs. Defining the ventilation contribution of each lung lobe as *λ_ROI_*_-i_, the right upper lung ventilation contribution *λ_ROI_*_-1_ was 25%, the right middle lung ventilation contribution *λ_ROI_*_-2_ was 10%, and the right lower lung ventilation contribution λ_*ROI*-3_ was 20%. The upper left lung ventilation contribution *λ_ROI_*_-4_ was 20% and the lower left lung ventilation contribution *λ_ROI_*_-5_ was 25%. Based on the characteristic changes in impedance signal amplitude due to regional lung lesions derived from 3-D simulation, the design (17) was used for regional ventilation assessment.

(17) $$\left\{ \begin{array}{l} FVC_{i}÷FVC=\alpha_{i}\lambda_{ROI-i}\\ \alpha_{i}\leq \omega \end{array}\right.$$

where *α_i_* is the regional ventilation contribution coefficient, the magnitude of which indicates the extent to which the actual contribution of ventilation change in the ith region of interest to the overall lung ventilation change differs from the ideal contribution. *ω* is the coefficient for evaluation of ventilation impairment, which is subsequently calibrated from the clinical trial data.

In addition, the traditional method of evaluating lung ventilation function utilizes the ratio of *FEV*1 and *FVC* in the whole lung area, and when the ratio is lower than 70%, it is defined as the presence of ventilation impairment in the lungs. Therefore, the ventilation parameters calculated from each lung lobe were designed (18).

(18) $$\left\{ \begin{array}{l} FEV1_{i}÷FVC_{i}=\beta_{i}\\ \beta_{i}\leq 70\% \end{array}\right.$$

where *β_i_* is the proportion of *FEV*1 of the ith region of interest to the *FVC* of the ith region of interest.

This article associates conventional evaluation methods and the characterization of changes in regional impedance amplitude to achieve pathological assessment of the ventilatory function of the five lung lobes. When any of the inequalities in Equations (17) and (18) are satisfied, the presence of ventilation impairment in that lobe is evaluated.

## 4. Experimental Design and Discussion of Results

### 4.1. Pentolung Lobar Ventilation Test System Construction

**Figure 14:**
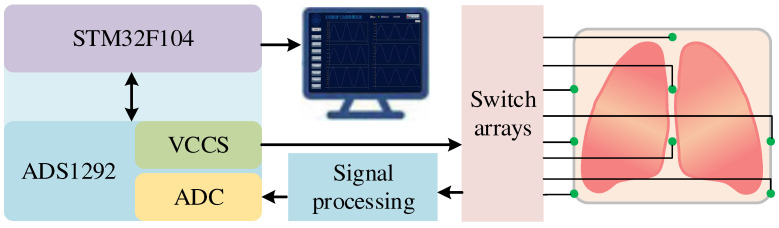
Detection system structure diagram.

In order to collect the regional impedance change data of five lung lobes at any respiratory moment in real time, a multi-channel human impedance acquisition system was designed, and its architecture is shown in Figure 14. The measurement system is mainly composed of a biosensing analog front-end ADS1292, a constant current source, a micro-processor unit (MCU), the switch array modules, the serial communication interface, and the signal processing unit. The current excitation with a frequency of 64 kHZ and an amplitude of 1 mA is injected into the human thoracic cavity by the ADS1292 built-in constant current source through a silver chloride electrode. The maximum size of impedance measurement noise that can be detected by the detection system is 15 mΩ. Then, when the amount of impedance change generated by the respiratory process exceeds 15 mΩ, the corresponding respiratory change can be detected. To ensure that the respiratory impedance curves of the five lung zones could be reconstructed, the sampling rate of the device was set to 2 KSPS. The voltage signal of the voltage measurement end is processed through filtering, differential amplification and demodulation to calculate the impedance data of the channel, and according to the MCU control of high-speed cross-switch action, the impedance data of the five lung lobes are collected synchronously, and the regional impedance data are transmitted to the upper computer in real time through the serial module, and the data preprocessing and parameter calculation algorithms are used to perform the operations such as waveform display, parameter calculation and data storage. The system can meet the demand of five-lung-lobe impedance synchronous detection.

**Figure 15:**
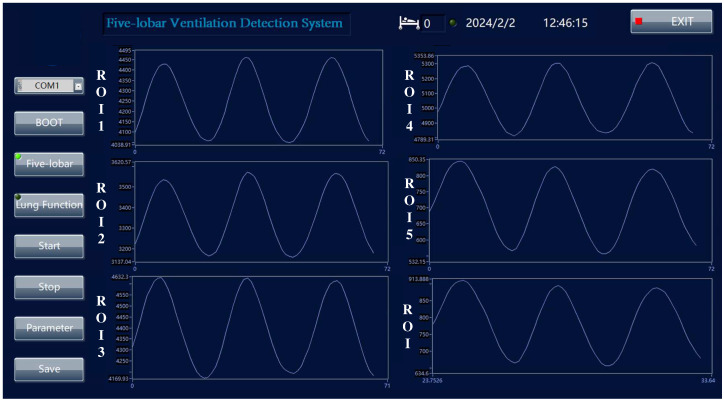
System working interface diagram.

In order to clearly and intuitively perform the data measurement of the transmission impedance in each measurement mode and to facilitate the recording, management, and analysis of real-time measurement data of different experimental individuals, the upper computer software was developed, and the upper computer interface is shown in Figure 15.

### 4.2. Experimental Flow Design

**Figure 16:**
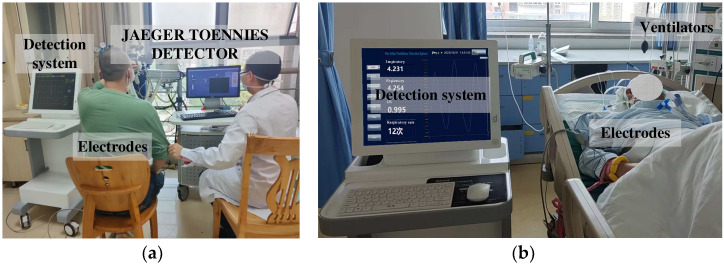
Test site diagram: (**a**) pulmonary function section tests; (**b**) critical care unit trials.

The clinical trial was divided into two parts: calibration of ventilation parameters and regional pathology assessment. For the five-lung-lobe ventilation parameter calculation, subjects were mainly selected from the pulmonary function department to carry out the test, and the Yeager lung function test instrument was used as the control instrument. The lung pathology assessment test was carried out mainly using patients with lung diseases, such as localized pulmonary fibrosis, from the critical care unit, and the test was carried out by means of a combined ventilator for patients in the rehabilitation stage. The data collection of this study was mainly carried out in tertiary hospitals, and its trial site diagram is shown in Figure 16a.

The accuracy of human thoracic impedance measurements is susceptible to interference from external factors, and therefore, the testing criteria for subjects need to be standardized when performing human trials. In order to minimize the influence of individual factors on the measurement data, subjects should avoid consuming drugs and foods that affect the level of lung function and ensure sufficient sleep two days before the test. The working electrodes were uniformly selected as commercially available disposable AgCl (silver chloride) electrocardiographic electrodes with an electrode diameter of 51 mm and an AC impedance of 3 KΩ or less. The specific standardized testing procedures were as follows:

1. The subject’s sitting posture was adjusted to ensure that the plane on which his or her upper body rests was perpendicular to the floor to minimize the effect of changes in the volume of air and blood in the lung tissue with changes in body posture. The subject’s clothing was also adjusted to ensure that there was no clothing contact on the surface of the chest and axillary body surface areas, and the position of the arms was controlled so that they did not come into contact with the skin of the chest body surface.
2. The intersection of the center of the first rib (labeled No. 8) and the center of the eighth rib (labeled No. 5) on the midclavicular line was found. Electrode positions No. 7 and No. 6 were marked equidistantly on the line between No. 8 and No. 5. The intersections of these four horizontal positions with the right axillary midline were labeled No. 1 through No. 4 from the bottom up, and the intersections with the left axillary midline were labeled No. 9 through No. 12 from the bottom up. Eventually, No. 1, No. 2, No. 3, No. 6, No. 7, No. 8, No. 9, and No. 10 were selected as the electrode placement points.
3. Medical alcohol was used to wipe the sweat and surface dirt at the electrode to be affixed, eight silver chloride electrodes were selected with the same specifications, and the electrode positions shown in Figure 16b were followed to ensure that the electrodes were in good contact with the surface of the skin to complete electrode affixation.
4. According to the patient category corresponding to the use of the German JAEGER company’s medical lung function tester JAEGER TOENNIES or ventilator at the same time to start the measurement, the switch array was used to complete the impedance of the five lobes of the lungs of the simultaneous measurement, continuous recording of a set of smooth breathing and a set of deep breathing of the subject data, and impedance data were entered into the database to complete the assessment of pulmonary ventilation function.

Following the test procedure described above, the average time required to complete a lung function test was 5 min per subject. This time has an absolute competitive advantage over traditional lung function testing machines.

### 4.3. Experimental Validation and Result Analysis

In order to verify the accuracy of the calculation of the five-lobe ventilation parameters, repeat experiments were carried out on 60 subjects according to the test specifications against the medical JAEGER TOENNIES lung function tester, and after completing a set of respiratory tests, each subject was kept in a state of relaxation for 10 min and then the testing process was repeated, and so on for each person until completing 20 sets of tests.

The data from the repeated trials for each subject were nonlinearly fitted according to (15). The results of fitting the data from 20 consecutive sets of repeated trials for one of the subjects are shown in Table 4. The fitting coefficients R^2^ were all greater than 0.995, which is close to 1, and the fit was good. SE-a, SE-b and SE-c in Table 4 are the standard errors of each regression parameter. It can be seen that the standard errors are all within 5%, indicating that the estimates of each regression parameter are good.

The matrix of characteristic coefficients *A* for this subject is obtained from (15) as shown in (19):

(19) $$A=\left[\begin{array}{ccc} 1.216\times 10^{5} & 7.127 & 218\\ 5.396\times 10^{4} & 6.343 & 104\\ 7.145\times 10^{4} & 6.563 & 153\\ 7.364\times 10^{4} & 6.728 & 172\\ 9.143\times 10^{5} & 6.343 & 192 \end{array}\right]$$

Based on the fitting effects of repeated trials with 60 subjects, it was found that the nonlinear fit of the five-lobe impedance data for a single individual was good, but the characteristic coefficient matrix *A* obtained from the fitting of different individuals differed significantly. To seek a model for calculating regional lobar ventilation parameters that can be used for different individuals, the individual factor with the greatest degree of influence was introduced to optimize the characteristic coefficient matrix *A*.

The single-test trial was conducted on 389 subjects and basic individual parameters such as height, weight, and age were selected and analyzed and found to have no significant correlation. Chest circumference and the ratio of longitudinal maximum thoracic spacing to transverse maximum thoracic spacing were further selected as specific parameters, and differences in spirometric changes were recorded synchronously, and a strong correlation was found between the ratio of longitudinal maximum thoracic spacing to transverse maximum thoracic spacing and pulmonary function parameters, which were then defined as *ξ*.

To further introduce a to optimize the eigencoefficient matrix *A*, the 389 subjects were divided into five groups according to *ξ*, and the elements of the eigencoefficient matrix *A* corresponding to the same *ξ* were fitted to obtain the set of correction coefficients, as shown in Table 5.

By fitting the column vector data corresponding to the eigen coefficient matrix A with different values of *ξ*, it is found that the monoexponential function gives the best fit. For any *ξ*, (20) always holds.

(20) $$\left\{ \begin{array}{l} a_{i}=m_{ai}\times \exp(-n_{ai}\times \xi )+k_{ai}\;\;\;\;\left\{i\in 1,2,3,4,5\right\}\\ b_{i}=m_{bi}\times \exp(-n_{bi}\times \xi )+k_{bi}\;\;\;\;\left\{i\in 1,2,3,4,5\right\}\\ c_{i}=m_{ci}\times \exp(-n_{ci}\times \xi )+k_{ci}\;\;\;\;\left\{i\in 1,2,3,4,5\right\} \end{array}\right.$$

In (20), *m* denotes the scale coefficient, *n* denotes the exponential coefficient, and *k* denotes the constant coefficient. The fitting relationship between the characteristic coefficients of different lung lobe regions and *ξ* is shown in Figure 16.

**Figure 17:**
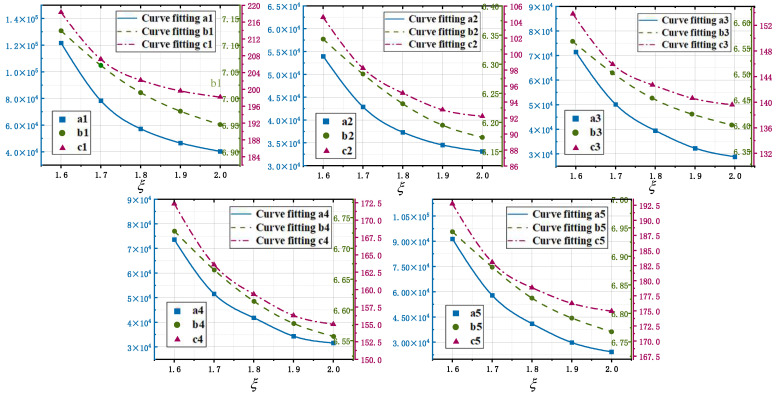
The fitting relationship between the fusion coefficient and *ξ*.

According to the fitting results in Figure 17, the correction coefficients in (20) are obtained for each correction coefficient, and then the correction parameter matrix, as shown in (21), can be obtained.

(21) $$\begin{array}{c} \left[\begin{array}{ccccccccc} m_{a1} & n_{a1} & k_{a1} & m_{b1} & n_{b1} & k_{b1} & m_{c1} & n_{c1} & k_{c1}\\ m_{a2} & n_{a2} & k_{a2} & m_{b2} & n_{b2} & k_{b2} & m_{c2} & n_{c2} & k_{c2}\\ m_{a3} & n_{a3} & k_{a3} & m_{b3} & n_{b3} & k_{b3} & m_{c3} & n_{c3} & k_{c3}\\ m_{a4} & n_{a4} & k_{a4} & m_{b4} & n_{b4} & k_{b4} & m_{c4} & n_{c4} & k_{c4}\\ m_{a5} & n_{a5} & k_{a5} & m_{b5} & n_{b5} & k_{b5} & m_{c5} & n_{c5} & k_{c5} \end{array}\right]\\ =\left[\begin{array}{ccccccccc} 5.15\times 10^{9} & 6.872 & 3.51\times 10^{4} & 38.25 & 3.151 & 6.881 & 1.96\times 10^{6} & 7.151 & 197\\ 1.38\times 10^{9} & 6.896 & 3.17\times 10^{4} & 30.68 & 3.018 & 6.098 & 2.87\times 10^{5} & 6.228 & 91\\ 5.52\times 10^{8} & 5.856 & 2.43\times 10^{4} & 55.84 & 3.466 & 6.345 & 1.38\times 10^{6} & 7.149 & 138\\ 1.08\times 10^{9} & 6.299 & 2.79\times 10^{4} & 43.71 & 3.257 & 6.491 & 2.57\times 10^{5} & 5.942 & 153\\ 8.67\times 10^{8} & 5.855 & 1.73\times 10^{4} & 33.24 & 3.053 & 6.692 & 1.28\times 10^{6} & 6.954 & 174 \end{array}\right] \end{array}$$

According to the modified parameter matrix of (21), the characteristic coefficient matrix *A* was optimized to form a five-lobe-based lung function parameter calculation method applicable to all individuals.

In order to verify the feasibility of the optimized calculation method, 20 trials were carried out again for validation in accordance with the standard test procedure, and the amount of impedance change in the five lobes of the lungs was synchronously recorded with the standard value of the standard pulmonary function tester, and the impedance dataset based on the human trials is shown in Table 6.

In this article, we verified the accuracy of the five-lobe ventilation calculation by comparatively analyzing the overall lung function parameters derived based on the five-lobe ventilation calculation. Then, the calculated values of lung function parameters FVCC and FEV1C were obtained by substituting the zonal impedance data in Table 6 into (16). Comparative analysis of the reference values of lung function parameters FVC and FEV1 recorded synchronously by the Yeager lung function tester, as shown in Figure 17, reveals that the calculated values of the lung function parameters obtained by the two methods are approximately equal to the standard values with a high accuracy rate.

In order to further verify the substitutability and consistency of this method with other methods, based on the optimized matrix of feature fusion coefficients *A*, a control test was carried out again on 10 subjects according to the same test specification, this time synchronously recording the measurement results of the medical lung function tester, and the results of the control test are shown in Table 7.

**Figure 18:**
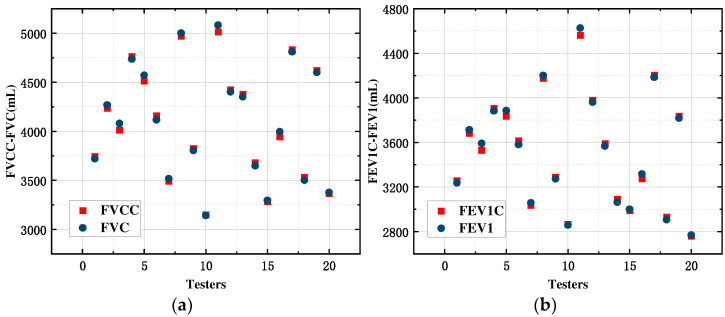
Comparison of calculated values with standard values. (**a**) FVCC-FVC; (**b**)FEV1C-FEV1.

According to the comparison data in Table 7, the relative errors of the corresponding parameters measured by the method in this paper were E1% and E2%, respectively, and the maximum values of E1 and E2 were 0.82% and 0.98%, respectively, which were less than 1%. Compared with the current method of calculating lung function parameters using lung biosensing information, the method presented in this manuscript has better accuracy. In addition, Figure 18 shows the comparison of the lung function parameters calculated by our test device with those obtained by a medical lung function tester, which can be seen to have a high degree of consistency compared with conventional instruments.

**Figure 19:**
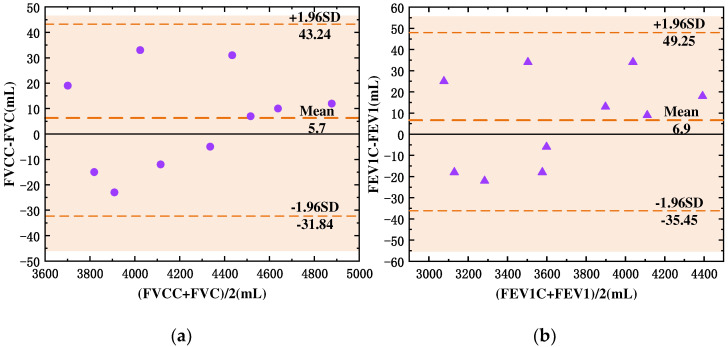
Bland-Altman scatter plot diagram of consistency analysis (**a**) Scattered point diagram about relation of FVCC and FVC; (**b**) scattered point diagram about relation of FEV1C and FEV1.

Considering the advantages of the Bland–Altman method for assessing the agreement between the two measurement techniques, it was chosen to verify the agreement of the calculated values with the standardized values based on the anthropometric data presented in Table 7. The Bland–Altman scatter plot is shown in Figure 19. All data points in Figure 19 are discretely distributed within the middle range of y = −1.96 SD and y = +1.96 SD, i.e., within the 95% confidence interval, indicating that the results of the two measurement methods are in good overall agreement. In addition, the mean deviation of FVCC vs. FVC was 5.7 mL, and the mean deviation of FEV1C vs. FEV1 was 6.9 mL, which was a negligible data deviation compared with its own deviation of 85 mL, and it had good consistency and strong substitution ability compared with the testing methods of medical lung function testing instruments.

Consistency analysis of pulmonary function parameters by the whole lung only validates the accuracy of the five-lobe ventilation calculation method from the summation point of view. To further assess the feasibility of five-lobe single-region ventilation calculation and pathological assessment, 50 patients with localized lung ventilation disorders were selected from the Respiratory and Critical Care Unit, and the amount of impedance change in the five lobes of the lungs and the amount of gas change in the lungs were measured synchronously with the combined ventilator. Substituting the data of 50 patients into (17) and (18), the *α_i_* and *β_i_* corresponding to each of the five lung lobes of each patient were calculated, and a 50 × 10 factor matrix was obtained. Based on the CT data of the 50 patients, the lobes with ventilation obstruction were labeled 1, and those with normal ventilation were labeled 0, enabling the formation of a 50 × 5 evaluation matrix consisting of five columns of binary variables.

**Figure 20:**
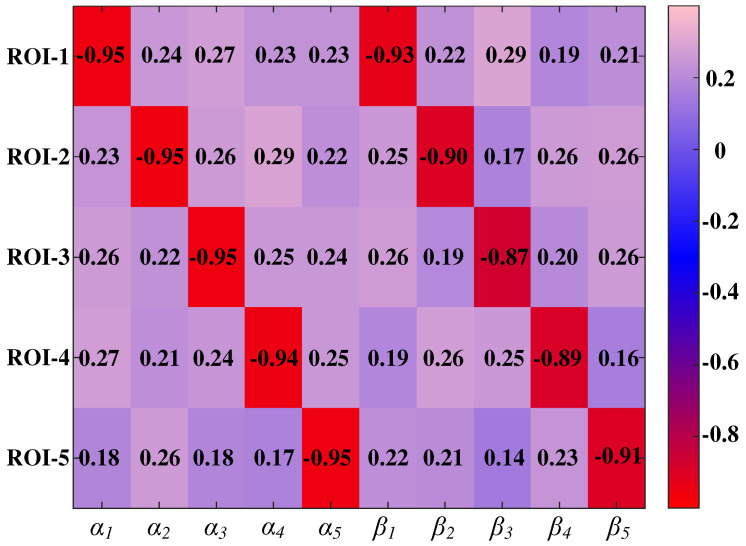
Heat map of point two column correlation coefficients.

To evaluate the correlation between the 10 factors and the occurrence of ventilation impairment in the five ROI zones, a point-two column correlation analysis was carried out for each column of the evaluation matrix using the factor matrix separately. The point-two column correlation coefficients were plotted on a heat map, as shown in Figure 20.

As can be seen from the individual correlation coefficients shown in Figure 20, for the ith binary variable of lung lobe ventilation impairment, the point two column correlation coefficients of the two variables, *α_i_* and *β_i_*, are very close to −1, indicating that there is a very strong negative correlation between them, and the variables evaluated for ventilation impairment. This means that when ventilation dysfunction occurs in the ith lung lobe, the value of its binary variable changes from 0 to 1, with a significant decrease in the values of the two continuous variables, *α_i_* and *β_i_*, thus assessing the lobe pathologic status. For the other lobes, the pointwise dichotomous correlation coefficients for the two variables *α* and *β* were close to 0, indicating a weak correlation. It was further demonstrated that the two indicators of lung pathology evaluation, *α_i_* and *β_i_*, designed for the data of impedance changes in specific lobes, can provide guidance for the assessment of ventilation disorders in specific lobes.

**Figure 21:**
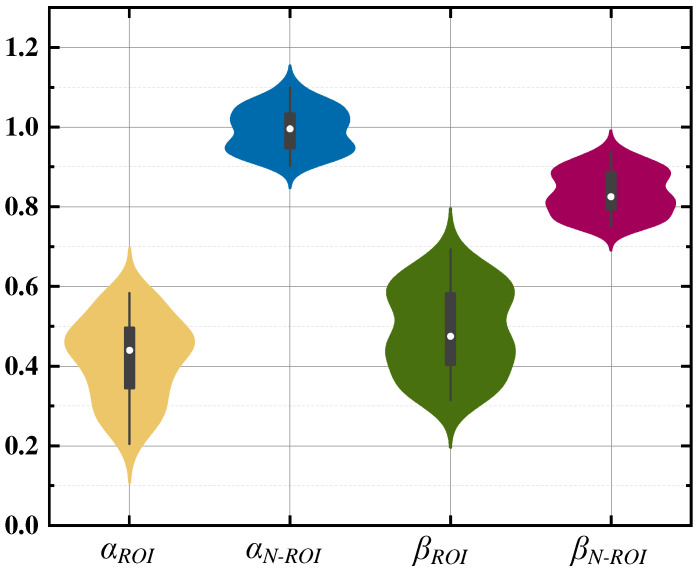
Distribution of violins.

In order to design the evaluation coefficient ω for ventilation impairment and to verify the feasibility of the *β* evaluation index, the violin distribution was plotted using data from 50 subjects, as shown in Figure 21. Where *α_ROI_* and *β_ROI_* represent the data distribution of *α_i_* and *β_i_* corresponding to the ROI when the ROI has lesions, and *α_N-ROI_* and *β_N-ROI_* represent the data distribution of *α_i_* and *β_i_* corresponding to the region of non-interest.

It can be seen that the maximum value of *α_i_* does not exceed 60% when a ventilation disorder occurs in the region of interest, and *ω* is set to 60% when no lesion occurs, and its value is in the range of [90%,110%]. Additionally, *β_i_* also satisfied (18) when lesions occurred in the ROI, and its values were all below 70%. Therefore, the method of evaluating ventilation impairment in the five lung lobes was determined using (22).

(22) $$\left\{ \begin{array}{l} \alpha_{i}\leq 60\%\\ \beta_{i}\leq 70\% \end{array}\right.$$

To further validate the feasibility of the pathology assessment method based on impedance sensing information of the five lung lobes, two patients, each with regional ventilation disorders occurring in specific lung lobes, for a total of ten subjects, were selected for a clinical trial. Pathologic assessment data were obtained, as shown in Table 8.

As can be seen in Table 8, the calculation results of the five-lobe regional ventilation barriers obtained from the evaluation indexes of (22) are the same as the evaluation results of CT imaging, which further validates the feasibility of the regional lung ventilation evaluation method based on the impedance sensing information of the five lobes of the lung.

## 5. Conclusions

In this article, a method for calculating pulmonary function parameters and assessing regional lung ventilation status based on impedance information of five lung lobes is proposed. The method realizes impedance-focused measurements in each lobe region by selecting the electrode pattern with the highest degree of interest in a specific lobe. This innovative approach more accurately characterizes the changes of impedance information in specific lung lobes during respiration, provides a feasible solution for the calculation of regional lung ventilation and state assessment, and improves the calculation accuracy of lung function parameters. On this basis, a corresponding detection system based on the biosensor analog front-end ADS1292 was designed and developed for clinical trials. Based on the measured data of 389 subjects in the pulmonary function testing department, the nonlinear equations between the gas changes and impedance changes of each lung lobe were constructed, and the coefficient matrix A was determined to obtain the parameter calculation equations. A secondary test was conducted to validate the method on 30 subjects, and the results showed that the maximum errors of FVC and FEV1 obtained by the method were 0.82% and 0.98%, respectively, which demonstrated a more excellent accuracy. Bland–Altman analysis of the synchronized measurement data showed good agreement of the method with the synchronized measurement data of medical spirometers. Based on the clinical five-lobe impedance measurement data of 50 critically ill patients, the evaluation index of five-lobe regional ventilation impairment was determined. Additionally, the validation test was carried out again in 10 cases. The results showed that the evaluation results of the method were consistent with the results of the CT image localization of the lesion area according to the structure of the lung lobes. Therefore, the method proposed in this article can not only be used for the detection of pulmonary function parameters in patients with various types of lung diseases to provide ventilation parameter support for each lung lobe but also has the ability to provide some preliminary evaluation for the localization of lung ventilation disorder foci.

In the follow-up work, in order to provide richer information on regional lung ventilation disorders, the amplitude–frequency characteristics of the impedance information of the five lung lobes corresponding to different degrees of lesions occurring in the lung region will be studied in depth, and the evaluation criteria and calculation model of the degree of lesions in the lung region will be constructed.

## Figures and Tables

**Table 1:** Conductivity parameter setting for each human tissue.

Tissues/Organs	Electrical Conductivity (S/m)
Skin and subcutaneous tissues	0.035
Blood layer	1.323
Muscle layer	0.479
Lung tissue	0.1802/*n*^0.1942^
Bioelectrodes	5.998 × 10^7^

**Table 2:** Optimal electrode configuration for pentapulmonary lobes.

ROI	Max P	Excitation	Measurement
Upper lobe of right lung	22.712	Electrode (2,7)	Electrode (3,8)
Middle lobe of right lung	18.733	Electrode (1,6)	Electrode (2,7)
Lower lobe of right lung	12.472	Electrode (1,9)	Electrode (2,6)
Upper lobe of left lung	29.542	Electrode (7,9)	Electrode (8,10)
Lower lobe of left lung	19.675	Electrode (6,9)	Electrode (7,10)

**Table 3:** Regional impedance-focusing measurement simulation dataset.

*n*	Z_1_ (Ω)	ΔZ_1_%	Z_2_ (Ω)	ΔZ_2_%	Z_3_ (Ω)	ΔZ_3_%	Z_4_ (Ω)	ΔZ_4_%	Z_5_ (Ω)	ΔZ_5_%
0.2	0.6864	0	0.7427	0	0.6363	0	0.8951	0	0.7427	0
0.25	0.7058	0.0283	0.7563	0.0182	0.6472	0.0171	0.9089	0.0155	0.7567	0.0188
0.3	0.7216	0.0512	0.7673	0.0331	0.6561	0.0311	0.9201	0.0281	0.7681	0.0341
0.35	0.7347	0.0704	0.7765	0.0453	0.6636	0.0429	0.9296	0.0387	0.7777	0.0471
0.4	0.7461	0.0867	0.7844	0.0561	0.6701	0.0531	0.9378	0.0478	0.7860	0.0582
0.45	0.7558	0.1011	0.7913	0.0654	0.6758	0.0620	0.9451	0.0558	0.7932	0.0680
0.5	0.7646	0.1138	0.7975	0.0737	0.6809	0.0701	0.9514	0.0631	0.7997	0.0767
0.55	0.7724	0.1253	0.8031	0.0813	0.6855	0.0773	0.9572	0.0695	0.8056	0.0846
0.6	0.7795	0.1357	0.8081	0.0880	0.6897	0.0839	0.9624	0.0753	0.8109	0.0918
0.65	0.7861	0.1451	0.8128	0.0943	0.6935	0.0899	0.9672	0.0807	0.8159	0.0984
0.7	0.7921	0.1539	0.8170	0.1001	0.6971	0.0955	0.9717	0.0856	0.8204	0.1045
0.75	0.7976	0.1619	0.8210	0.1053	0.7004	0.1007	0.9758	0.0903	0.8246	0.1102
0.8	0.8027	0.1694	0.8247	0.1103	0.7035	0.1056	0.9796	0.0946	0.8285	0.1154
0.85	0.8076	0.1765	0.8282	0.1150	0.7064	0.1101	0.9832	0.0986	0.8322	0.1204
0.9	0.8121	0.1831	0.8314	0.1194	0.7091	0.1144	0.9866	0.1024	0.8357	0.1251
0.95	0.8164	0.1893	0.8345	0.1235	0.7117	0.1185	0.9898	0.1059	0.8390	0.1295
1	0.8204	0.1951	0.8374	0.1274	0.7142	0.1223	0.9928	0.1093	0.8421	0.1336
1.05	0.8242	0.2007	0.8401	0.1311	0.7165	0.1261	0.9957	0.1125	0.8450	0.1376
1.1	0.8278	0.2060	0.8428	0.1346	0.7187	0.1295	0.9984	0.1156	0.8478	0.1414
1.15	0.8313	0.2110	0.8453	0.1380	0.7208	0.1329	1.0010	0.1184	0.8505	0.1450
1.2	0.8345	0.2158	0.8476	0.1412	0.7229	0.1361	1.0034	0.1212	0.8531	0.1484
1.25	0.8377	0.2204	0.8499	0.1443	0.7248	0.1391	1.0061	0.1240	0.8555	0.1518
1.3	0.8407	0.2247	0.8521	0.1473	0.7266	0.1420	1.0081	0.1264	0.8578	0.1549
1.35	0.8436	0.2289	0.8542	0.1501	0.7285	0.1448	1.0103	0.1288	0.8601	0.1580
1.4	0.8464	0.2330	0.8563	0.1528	0.7302	0.1475	1.0125	0.1313	0.8623	0.1609

**Table 4:** Table of fitting results of 20 sets of repetitive tests for a single individual.

ROI	a	SE-a	b	SE-b	c	SE-c	R^2^
ROI-1	1.216 × 10^5^	2.795 × 10^3^	7.127	0.192	218	7.194	0.99738
ROI-2	5.396 × 10^4^	9.173 × 10^2^	6.343	0.027	104	3.016	0.99653
ROI-3	7.145 × 10^4^	1.512 × 10^3^	6.563	0.126	153	3.273	0.99564
ROI-4	7.364 × 10^4^	8.823 × 10^2^	6.728	0.105	172	3.027	0.99523
ROI-5	9.143 × 10^4^	1.737 × 10^3^	6.943	0.217	192	2.936	0.99842

**Table 5:** Set of correction coefficients based on human testing.

*ξ*	ROI	a	b	c	R^2^
1.6	1	1.216 × 10^5^	7.127	218.317	0.9993
	2	5.396 × 10^5^	6.343	104.579	0.9982
	3	7.145 × 10^5^	6.563	153.781	0.9965
	4	7.364 × 10^4^	6.728	172.361	0.9979
	5	9.143 × 10^4^	6.943	192.748	0.9986
1.7	1	7.828 × 10^4^	7.062	207.133	0.9988
	2	4.287 × 10^5^	6.283	98.218	0.9964
	3	5.015 × 10^4^	6.502	145.903	0.9977
	4	5.156 × 10^4^	6.665	163.592	0.9991
	5	5.784 × 10^4^	6.881	183.057	0.9993
1.8	1	5.715 × 10^4^	7.011	202.575	0.9973
	2	3.732 × 10^5^	6.232	95.107	0.9981
	3	3.945 × 10^4^	6.453	142.692	0.9965
	4	4.183 × 10^4^	6.614	159.347	0.9963
	5	4.105 × 10^4^	6.827	178.849	0.9971
1.9	1	4.656 × 10^4^	6.976	199.623	0.9987
	2	3.453 × 10^5^	6.195	93.016	0.9983
	3	3.231 × 10^4^	6.422	140.613	0.9972
	4	3.437 × 10^4^	6.578	156.278	0.9974
	5	2.984 × 10^4^	6.792	176.243	0.9974
2.0	1	4.029 × 10^4^	6.951	198.174	0.9976
	2	3.312 × 10^5^	6.174	92.213	0.9987
	3	2.883 × 10^4^	6.401	139.592	0.9980
	4	3.158 × 10^4^	6.557	155.034	0.9971
	5	2.426 × 10^4^	6.768	174.964	0.9969

**Table 6:** Impedance datasets based on human trials.

*ξ*	ΔZ_1_ (Ω)		ΔZ_2_ (Ω)		ΔZ_3_ (Ω)		ΔZ_4_ (Ω)		ΔZ_5_ (Ω)	
	ΔZ_1FVC_	ΔZ_1FEV1_	ΔZ_2FVC_	ΔZ_2FEV1_	ΔZ_3FVC_	ΔZ_3FEV1_	ΔZ_4FVC_	ΔZ_4FEV1_	ΔZ_5FVC_	ΔZ_5FEV1_
1.7	0.51542	0.50224	0.45696	0.44353	0.50108	0.48751	0.5147	0.5010	0.52705	0.51355
1.6	0.49771	0.48538	0.45417	0.44123	0.48894	0.47608	0.50287	0.4899	0.50754	0.49491
1.7	0.52187	0.50988	0.46354	0.45132	0.50777	0.49537	0.52142	0.5089	0.53369	0.52137
1.8	0.56098	0.54176	0.48845	0.46937	0.54387	0.52401	0.55622	0.5363	0.57899	0.55899
1.7	0.53277	0.51765	0.47465	0.45924	0.51907	0.5034	0.53279	0.5170	0.54492	0.52935
1.8	0.54785	0.53428	0.47541	0.46194	0.53029	0.51631	0.54263	0.5286	0.56531	0.55122
1.6	0.48058	0.46792	0.43618	0.42286	0.47109	0.45799	0.48487	0.4716	0.49001	0.47713
1.9	0.58102	0.56367	0.4971	0.48021	0.5624	0.54448	0.57276	0.5549	0.60732	0.58901
1.9	0.55491	0.53964	0.47169	0.45684	0.53547	0.51983	0.54592	0.5303	0.57979	0.56377
1.8	0.52059	0.5111	0.44836	0.43894	0.5023	0.49263	0.51456	0.5048	0.53707	0.5273
1.6	0.51245	0.50424	0.46965	0.46102	0.50441	0.49578	0.51844	0.5097	0.5227	0.51424
2.0	0.57874	0.56801	0.48779	0.47748	0.55955	0.54849	0.56805	0.5571	0.61257	0.60111
1.8	0.55287	0.53354	0.48039	0.46121	0.53547	0.51556	0.54782	0.5278	0.57053	0.55046
2.0	0.56001	0.54193	0.4698	0.45245	0.54027	0.52179	0.5489	0.5305	0.5926	0.57341
1.6	0.47506	0.46643	0.43038	0.4213	0.46537	0.45646	0.47909	0.4701	0.48439	0.47562
1.9	0.55801	0.53918	0.4747	0.45639	0.53865	0.51936	0.5491	0.5298	0.58305	0.56329
1.6	0.50927	0.49708	0.46631	0.45351	0.50106	0.48828	0.51507	0.5022	0.51942	0.50689
1.8	0.53193	0.51335	0.45961	0.44118	0.5139	0.49492	0.5262	0.5072	0.54879	0.52962
2.0	0.58318	0.56422	0.49205	0.47384	0.56414	0.54459	0.5726	0.5532	0.61732	0.59708
1.7	0.50535	0.4862	0.4467	0.42715	0.49071	0.47109	0.50425	0.4845	0.51673	0.4972

**Table 7:** Controlled trial results.

Testers	FVC	FEV1	FVCC	FEV1C	E1%	E2%
1	4512	4106	4519	4115	0.17	0.23
2	4121	3586	4109	3568	−0.29	−0.48
3	3692	3064	3711	3089	0.52	0.83
4	3921	3294	3898	3272	−0.57	−0.66
5	4338	3601	4333	3595	−0.11	−0.17
6	4007	3487	4040	3521	0.82	0.98
7	4871	4384	4883	4402	0.26	0.42
8	3827	3138	3812	3120	−0.39	−0.57
9	4633	3892	4643	3905	0.21	0.33
10	4418	4021	4449	4055	0.72	0.85

**Table 8:** Pathological assessment results.

No.	CT	Text	α_1_	α_2_	α_3_	α_4_	α_5_	β_1_	β_2_	β_3_	β_4_	β_5_
1	ROI-2	ROI-2	103%	27%	100%	86%	33%	0.86	0.33	0.79	0.82	0.85
2	ROI-3	ROI-3	105%	106%	41%	103%	106%	0.92	0.72	0.56	0.91	0.82
3	ROI-5	ROI-5	103%	102%	108%	109%	52%	0.91	0.85	0.82	0.76	0.31
4	ROI-2	ROI-2	106%	43%	105%	102%	104%	0.88	0.42	0.83	0.87	0.86
5	ROI-1	ROI-1	38%	104%	107%	109%	103%	0.46	0.75	0.82	0.79	0.81
6	ROI-4	ROI-4	107%	105%	106%	50%	99%	0.93	0.87	0.79	0.45	0.75
7	ROI-5	ROI-5	107%	101%	106%	104%	0.49	0.93	0.89	0.86	0.81	0.49
8	ROI-1	ROI-1	55%	105%	102%	104%	1.01	0.62	0.9	0.91	0.93	0.91
9	ROI-4	ROI-4	101%	105%	103%	43%	1.07	0.88	0.83	0.77	0.42	0.72
10	ROI-3	ROI-3	104%	110%	58%	101%	0.98	0.78	0.8	0.4	0.86	0.93

## Data Availability

The data that support the findings of this study are available from the corresponding author upon reasonable request.

## Nomenclature

The following abbreviations and symbols are mainly used in this manuscript:

FVC	Forced ventilation capacity
FEV1	Forced expiratory volume in one second
ROI	Region of interest
σ_i_	The conductivity of the ith discrete region
*S*	The sensitivity matrix
Δ*L*	The total air changes in the lungs
Δ*L_ROI-i_*	The volume of air change in the ith ROI
Zi	The impedance value of the ith ROI region
ΔZi	The amount of impedance change in the i-th ROI region
*A*	The coefficient matrix
ξ	The ratio of the maximum longitudinal distance of the thoracic cavity to the maximum distance of the transverse cavity
FVCi	Forced ventilation volume for the ith ROI
FEV1i	One-second ventilation for the ith ROI
FVCC	The calculation of forced ventilation capacity
FEV1C	The calculation of forced expiratory volume in one second
α_i_	The ratio of ventilation in the ith ROI region to the overall ventilation
β_i_	The ratio of one-second ventilation to forced ventilation for the ith ROI
